# Fatigue Crack Growth Behavior of CP-Ti Cruciform Specimens with Mixed Mode I-II Crack under Biaxial Loading

**DOI:** 10.3390/ma15051926

**Published:** 2022-03-04

**Authors:** Jia-Yu Liu, Wen-Jie Bao, Jia-Yu Zhao, Chang-Yu Zhou

**Affiliations:** 1 School of Mechanical and Power Engineering, Nanjing Tech University, Nanjing 211816, China; 201961207109@njtech.edu.cn (J.-Y.L.); 202161107023@njtech.edu.cn (W.-J.B.); 201961207106@njtech.edu.cn (J.-Y.Z.); 2 Jiangsu Key Lab of Design and Manufacture of Extreme Pressure Equipment, Nanjing Tech University, Nanjing 211816, China

**Keywords:** CP-Ti, fatigue crack growth, mixed mode I-II crack, biaxial load ratio, crack inclination angle

## Abstract

Investigations on the fatigue crack growth of commercial pure titanium are carried out with cruciform specimens under different biaxial load ratios (*λ* = 0, 0.5, and 1) and crack inclination angles (*β* = 90°, 60°, and 45°) in this paper. Based on the finite element results, the modified solution of stress intensity factors *K_I_* and *K_II_* for cruciform specimens containing mixed mode I-II crack is obtained by considering crack size, biaxial load ratio, and crack inclination angles. The experimental results show that the maximum tangential stress criterion is fit for the prediction of crack initiation angles for mixed model I-II crack under uniaxial or biaxial loading condition. When the biaxial load ratio increases, the crack propagation angle becomes smaller, and so does the fatigue crack growth rate of mode I crack or mixed mode I-II crack. Based on an equivalent stress intensity factor, a new valid stress intensity factor is proposed to better describe the biaxial fatigue crack growth behavior, which can demonstrate the contribution of mode I and mode II of stress intensity factor.

## 1. Introduction

In engineering, crack damage in structures or components is often under the condition of complex stress. Commercial pure titanium (CP-Ti) is widely used in pressure vessels due to its good corrosion resistance and machinable deformation properties. In order to ensure the safety of titanium pressure vessels, an in-depth understanding of the fracture behavior of mixed mode crack is required. Uniaxial fatigue crack growth models are inadequate for mixed mode complex stress situations, so it is necessary to study the fatigue crack growth under biaxial loading.

There have been many studies related to uniaxial fatigue crack growth, which are concerned with the uniaxial fatigue crack growth of AZ31B magnesium alloy [1,2], 7075-T651 aluminum alloy [3], CP-Ti based metal, and weldment [4,5].

Studies on the growth of uniaxial mixed mode I-II fatigue cracks have been referenced by previous work [6,7,8,9]. They applied the maximum tangential stress (MTS) criterion to investigate the propagation direction of a mixed mode I-II crack.

Cruciform specimens are widely used in many laboratories and research institutions for biaxial fatigue crack growth tests [10,11]. By changing the angle of the initial crack and different load ratios, a mode I or mixed mode I-II crack can be achieved, and a uniform stress field can be distributed in the central region [12]. Liu and Dittmer [13] studied the fatigue crack growth behavior of aluminum alloys at different biaxial load ratios, and they found that the direction and crack growth rate were controlled by larger biaxial load components, and the stress parallel to the crack had a negligible effect on the crack growth rate. This was not the case for Yuuki [14], who showed that the effect of biaxial ratio on the crack growth rate was negligible at lower stress levels only, but at higher stress levels, the fatigue crack growth rate was significantly higher under a biaxial ratio of −1 than that under biaxial load ratios of 0 and 1. Hopper and Miller [15] suggested that stresses parallel to the crack would make crack growth rate decrease. Lee and Taylor [16] reported that the fatigue crack life became shorter as the biaxial stress ratio increased, but the exact crack growth rate and crack driving force data were not given in their paper to support the conclusion. In a study reported by Anderson [17], it was found that the stress field of biaxial loading caused a change of fatigue crack growth rate, and that equal biaxial tension resulted in a lower crack growth rate compared to uniaxial loading. Joshi and Shewchuk [18] used bending loaded specimens to investigate biaxial fatigue crack growth, and the paper only mentioned that the crack growth rate was affected by biaxial load ratio, but did not specify what kind of effect. Sunder [19] concluded that the stress biaxial load ratio had a significant effect on the fatigue crack growth rate, and the growth rate was sensitive to the change of biaxiality, with an increase of biaxial ratio leading to a decrease of crack growth rate. Meng [20] investigated the effect of phase difference and stress ratio on biaxial fatigue crack growth of ZK60 magnesium alloy and found that the crack growth mechanism was the same at different stress ratios, but the growth mechanism was different with phase differences. Shlyannikov [21] found that cruciform specimens with a thinned central observation zone were more convenient for studying the crack growth rate under biaxial loading, and suggested that fatigue crack growth with a mixed mode crack might be influenced more by plastic stress intensity factor.

Compared to uniaxial loading, biaxial fatigue crack growth still holds a lot of promise for research. More previous scholars’ studies on biaxial fatigue crack growth are focused on alloys used in aerospace, and the conclusions are different due to the different materials of cruciform specimens. In this paper a CP-Ti cruciform specimen is used, and the modified solutions of stress intensity factors *K_I_* and *K_II_* for cruciform specimens containing mixed mode I-II cracks are obtained through finite element simulation. Furthermore, fatigue crack growth rates under different biaxial load ratios and crack inclination angles based on tests are discussed, and the effects of stress intensity factors *K_I_*, *K_II_* on fatigue crack growth rate under different biaxial load ratios are analyzed. Finally, a new equivalent stress intensity factor is proposed to describe the biaxial crack growth behavior of CP-Ti.

## 2. Biaxial Fatigue Crack Growth Test

### 2.1. Material

The material of the cruciform specimen is CP-Ti (Baose, Nanjing, China). The mechanical properties are shown in Table 1 and its chemical compositions are shown in Table 2.

### 2.2. Cruciform Specimen and Test Methods

**Figure 1:**
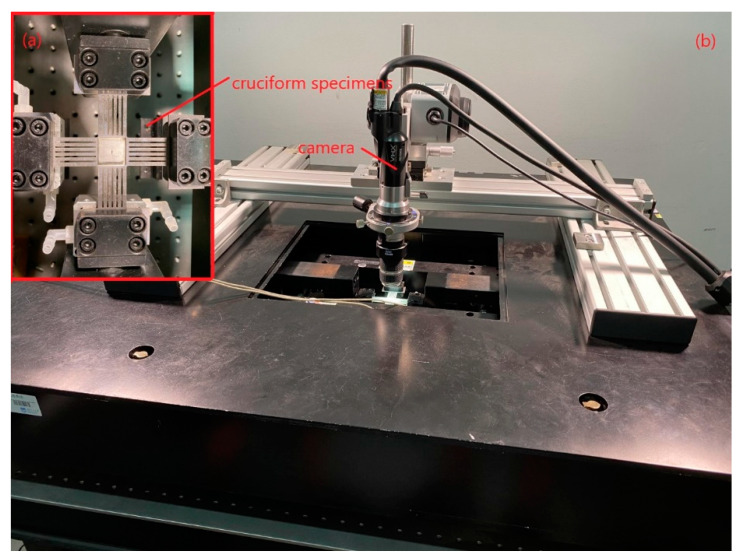
Biaxial test machine and cruciform specimen. (**a**) Specimen clamping diagram (**b**) Test Machines.

**Figure 2:**
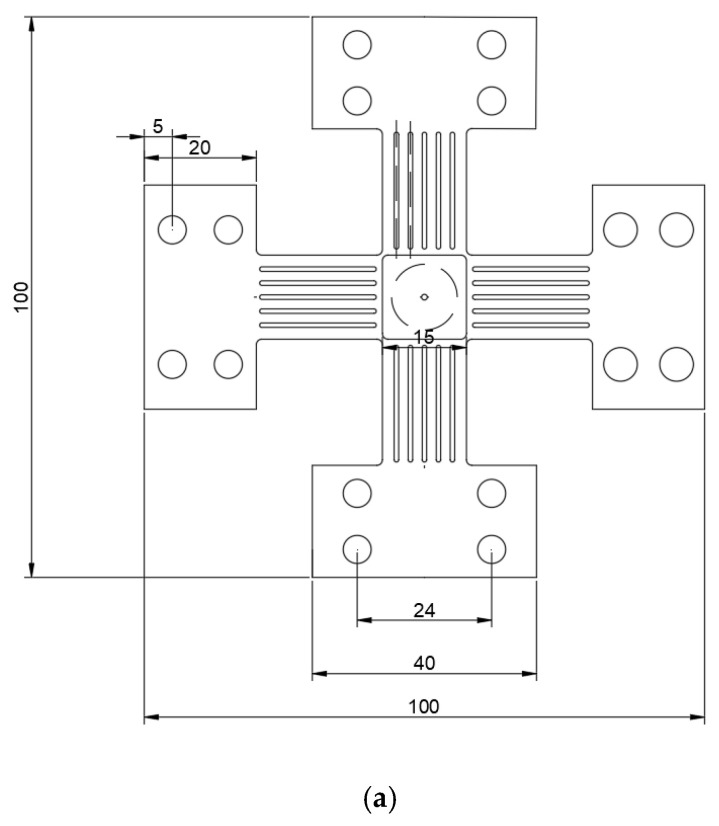

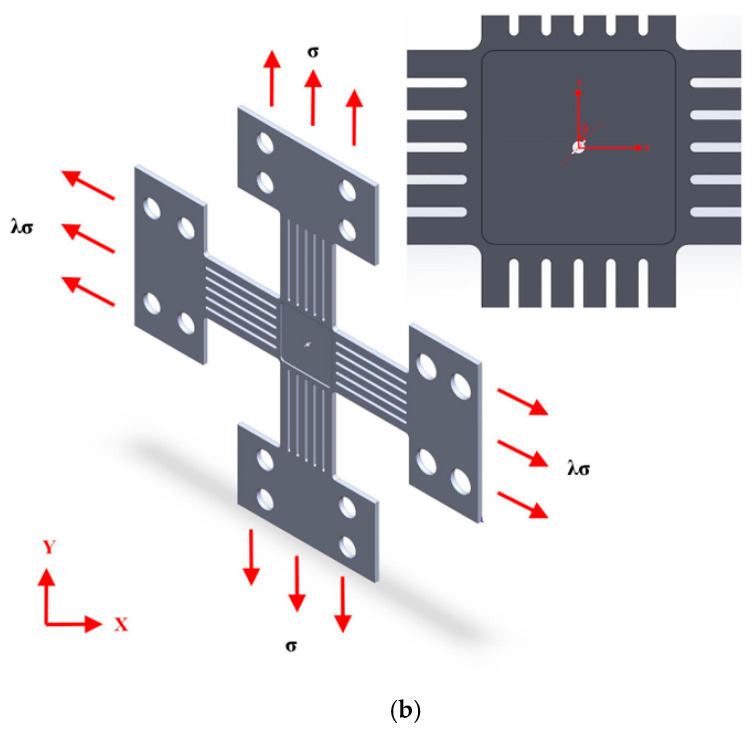
The cruciform specimen. (**a**) Dimension of cruciform specimen and (**b**) three-dimensional specimen (*λ* = 0, 0.5, and 1). σ is the load in the Y direction, *λσ* is the load in the X direction.

An IPBF-5000 series test machine of Tianjin Kair Measurement and Control Company (Tianjin, China) is used (Figure 1) [22], and the specimen is shown in Figure 2. A loading frequency of 0.2 Hz is used for all experiments, and the waveform is a sine wave. The maximum load in the vertical direction (*Y*-axis) is 270 MPa as a constant, the load in the horizontal direction (*X*-axis) is changed according to biaxial load ratio *λ*, *λ* (*λ* = *σx*/*σy*) with cyclic stress ratio *R* = 0 and crack inclination angles of 90°, 60°, and 45°. Before the fatigue crack growth test, the crack needs to be prefabricated. For the crack with inclination angle of 90°, the load perpendicular to the crack (*Y*-axis) is applied to propagate the crack; it should be noted that the maximum stress intensity factor for the pre-crack must be less than the equivalent force intensity factor of the subsequent mixed mode I-II mode crack [23]. For the inclined cracks of 60° and 45°, equal biaxial loading (*λ* = 1) for pre-cracking is performed [16], which is to eliminate the effect of machined notch on the crack growth. In this paper, the fatigue crack growth is studied under biaxial load ratios of 0.5, 1, and 0 (uniaxial load) with crack inclination angles of 90°, 60°, and 45°.

The overall dimensions of the specimen are 100 × 100 (mm), with a thickness of 1.2 mm, thinned to 0.4 mm in the center, and a 15 × 15 (mm) square in the central observation area. Before the test, the central observation area of the specimen needs to be sanded with 400 to 2000 grit sandpaper (Kraftwell, Hangzhou, China) in turn to make the central observation area as smooth as possible to facilitate the observation of crack growth and measurement.

## 3. Solution of Stress Intensity Factor for Cruciform Specimen

The stress intensity factor is an important parameter to characterize fatigue crack growth, and is a quantitative expression of stress level at crack tip, which is the driving force for crack growth. Therefore, an accurate solution of the stress intensity factor is of great importance for fatigue crack growth. In general, for biaxially loaded specimens, the stress intensity factor calculation Formulas (1) and (2) of the infinite plate can be used [21]. There are differences between the stress intensity factors of the cruciform specimen used in this article and the infinite plate, therefore, the stress intensity factor suitable for the mixed mode I-II fracture for the cruciform specimen must be derived.

(1) $$K_{I}=\sigma \sqrt{\pi a}\left(\lambda \cos^{2}\beta +\sin^{2}\beta \right)$$

(2) $$K_{II}=\sigma \sqrt{\pi a}\left(1-\lambda \right)\cos\beta \sin\beta$$

**Figure 3:**
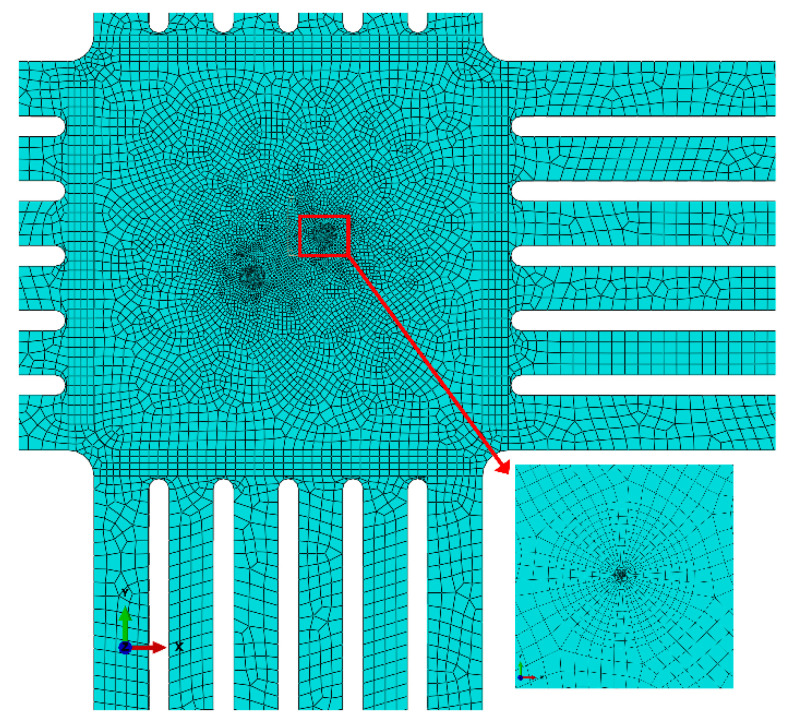
Finite element model of cruciform specimen with mesh and refined mesh near crack.

In this paper, ABAQUS6.14 software (Paris, France) is used to calculate the stress intensity factors *K_I_* and *K_II_* for the cruciform specimen model. A range of radius 0.5 is selected at the tip of crack and set as a wedge-shaped mesh to refine it. The finite element model of cruciform specimen and the surrounding meshes are shown in Figure 3. The entire model uses the C3D8R element type. The number of elements in the model is 36,925 and the number of nodes is 58,202. We found that the results of finite element simulation are independent of the mesh size when the crack tip mesh size is less than 0.05 mm. Therefore, the mesh size within 0.5 mm of the crack tip is divided equally by 0.05 mm and the mesh size outside the 0.5 mm range gradually increases.

### 3.1. Stress Intensity Factor for Cruciform Specimen

#### 3.1.1. Stress Intensity Factor for Mode I Crack (β = 90°)

**Figure 4:**
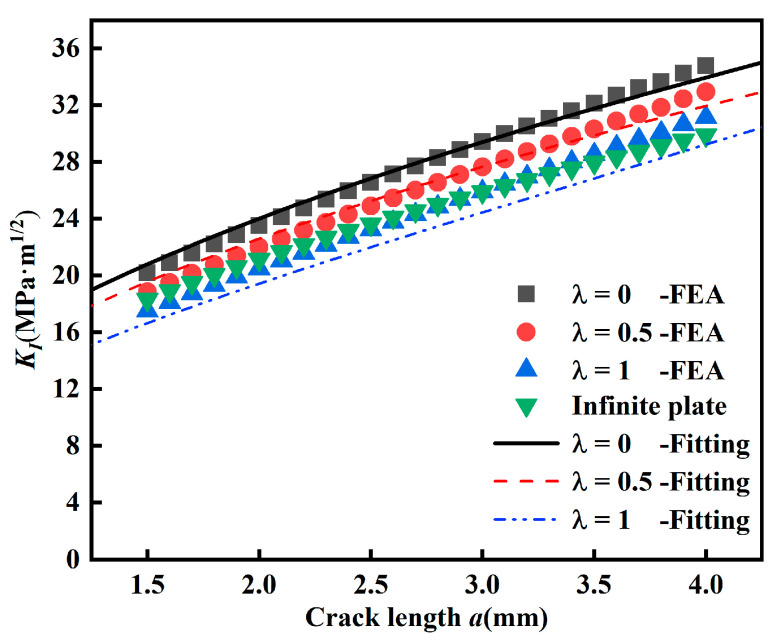
Relationship of *K_I_* with crack length *a*.

In Figure 4, the finite element solutions of *K_I_* for mode I cracks (*β* = 90°) are compared with *K_I_* of the infinite plate. It can be seen that the stress intensity factor *K_I_* of the infinite plate is independent of biaxial load ratio, while the stress intensity factor *K_I_* of the cruciform specimen is decreasing with horizontal load, which is related to biaxial load ratio *λ*. Obviously, the stress intensity factor *K_I_* of the infinite plate is not suitable for the *K_I_* of the cruciform specimen under biaxial loading.

#### 3.1.2. Stress Intensity Factor for Mixed Mode I-II Crack (β = 60°, 45°)

**Figure 5:**
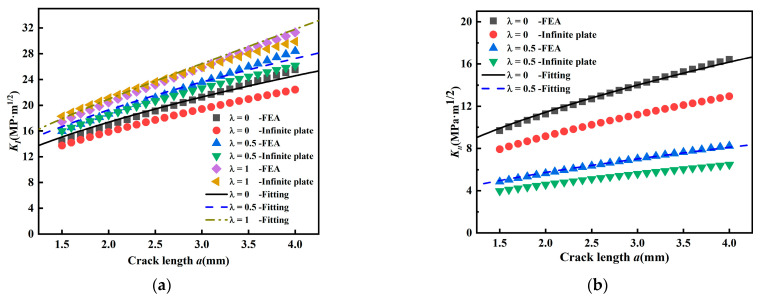
Stress intensity factor versus crack length *a* (*β* = 60°). (**a**) Relationship between *K_I_* and *a* and (**b**) relationship between *K_II_* and *a*.

Figure 5 shows the comparison of finite element solutions of stress intensity factor *K_I_* and *K_II_* with those solutions for infinite plate when crack inclination angle *β* = 60°. From Figure 5, it can be seen that *K_I_* becomes larger and *K_II_* becomes smaller as biaxial load ratio increases. When *λ* = 1, *K_II_* disappears and only *K_I_* exists. As seen in Figure 5a, there is a certain error between the *K_I_* solution by finite element and the *K_I_* solution of infinite plate. From Figure 5b, the difference between the finite element solution of *K_II_* and the *K_II_* solution of the infinite plate is greater. *K_II_* can cause dislocation movement based on planar slip or wavy slip [11].

From Figure 5, it can be seen that there is a certain error between the infinite flat plate solution and the finite element solution, and it increases with the crack length. This is because the ratio of crack length *a* to the width of the finite region is smaller when the crack is shorter, which is similar to the case of the crack in the infinite flat plate. However, as the crack propagates, the crack length *a* in the finite element model is more and more influenced by the shape of the specimen, so the error becomes larger. Considering the crack propagation angle *β*, biaxial ratio *λ*, and the effect of geometric factor *a/w*, the infinite flat plate solution is modified to reduce the error.

**Figure 6:**
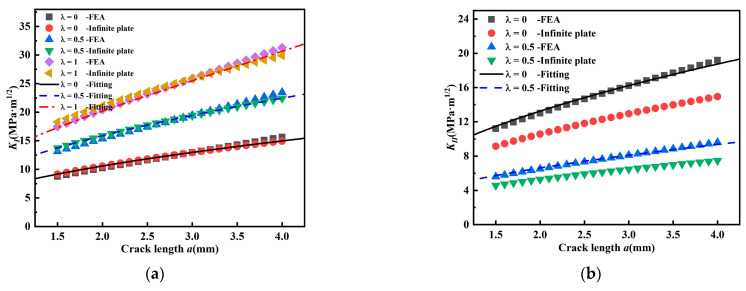
Stress intensity factor *K_I_* and *K_II_* (*β* = 45°). (**a**) Relationship between *K_I_* and *a* and (**b**) relationship between *K_II_* and *a*.

Figure 6 shows the stress intensity factors *K_I_* and *K_II_* for *β* = 45°; and the variation regularity is similar to Figure 5 and the stress intensity factor *K_II_* also decreases with biaxial load ratio. However, in the uniaxial load (*λ* = 0) state, the overall level of *K_I_* at the same crack length is lower than that at *β* = 60°, and *K_II_* is higher than that at *β* = 60°. The difference between the two solutions of *K_I_* at 45 degrees is smaller compared *K_I_* at 60 degrees, and it can be concluded that the inclined crack is less influenced by geometric factors and biaxial ratio *λ* at 45 degrees.

Based on the finite element solution of the stress intensity factor, it is found that it is not enough to consider the influence of geometric factors of the cruciform specimen only, but also the influence of biaxial load ratio *λ* and crack inclination angle *β*. Therefore, the correction parameter *f(λ,β)* is introduced. The correction coefficients of Shanyanskiy [24] are referred for the cruciform specimens *K_I_* and *K_II_* (Equations (3)–(7)).

(3) $$K_{I}=Y_{I}\left(a/w\right)\times f\left(\lambda ,\beta \right)\times \sigma \times \sqrt{\pi a}\times \left(\lambda \cos^{2}\beta +\sin^{2}\beta \right)$$

(4) $$K_{II}=Y_{II}\left(a/w\right)\times f\left(\lambda ,\beta \right)\times \sigma \times \sqrt{\pi a}\times \left(1-\lambda \right)\cos\beta \sin\beta$$

(5) $$Y_{I}=A_{I}+B_{I}\times \left(a/w\right)+C_{I}\times {\left(a/w\right)}^{2}$$

(6) $$Y_{II}=A_{II}+B_{II}\times \left(a/w\right)+C_{II}\times {\left(a/w\right)}^{2}$$

(7) $$f\left(\lambda ,\beta \right)=\frac{1}{1+C\lambda \sin\beta }$$

where *w* is width of thinned area of cruciform specimen, *Y_I_(a/w)* and *Y_II_(a/w)* are shape factors, and *f(λ,β)* is biaxial loading factor.

(8) $$\begin{array}{c} Y_{I}\left(a/w\right)=0.9624+0.05348\times \left(a/w\right)+1.22734\times {\left(a/w\right)}^{2}\\ Y_{II}\left(a/w\right)=1.19956+0.21922\times \left(a/w\right)+0.17017{\left(a/w\right)}^{2} \end{array}\}$$

(9) $$f\left(\lambda ,\beta \right)=\frac{1}{1+0.05287\times \lambda \times \sin\beta }$$

The comprehensive error analysis shows that the average error between the finite element solution and the fitted formula solution is 3%, and the maximum error is less than 7%. Therefore, the fitted formula can be used for the calculation of stress intensity factor of cruciform specimen.

## 4. Crack Propagation Angle

For the MTS criterion of brittle fracture, it is assumed that the crack propagates along a path perpendicular to the direction of maximum tension stress. This criterion is proved to have a high consistency with the data under tensile experiments and used as a common method for scholars to study the crack propagation angle.

In general, biaxial fatigue crack growth is determined by the following three parameters: stress biaxial ratio *λ*, crack inclination angle *β*, and stress intensity factor *K_I_* and *K_II_*. During crack growth, a mixed mode I-II crack may occur.

Based on MTS, Erdogan and Sih [25] proposed Equation (10) to calculate the crack propagation angle *θ*_0_.

(10) $$\theta_{0}=2\arctan\left(\frac{1\pm \sqrt{1+8{\left(\frac{K_{II}}{K_{I}}\right)}^{2}}}{4\frac{K_{II}}{K_{I}}}\right)$$

where the predicted angle here is relative to crack surface, and *θ*_0_ = 0 indicates that the crack extends along a straight line. When *K_II_* > 0, *θ*_0_ < 0. When *K_II_* < 0, *θ*_0_ > 0.

**Figure 7:**
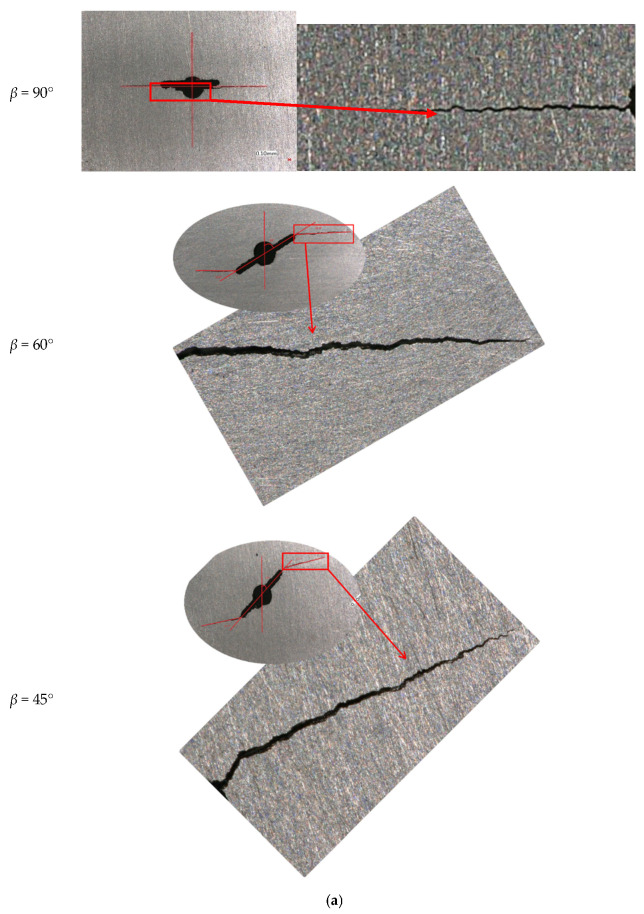

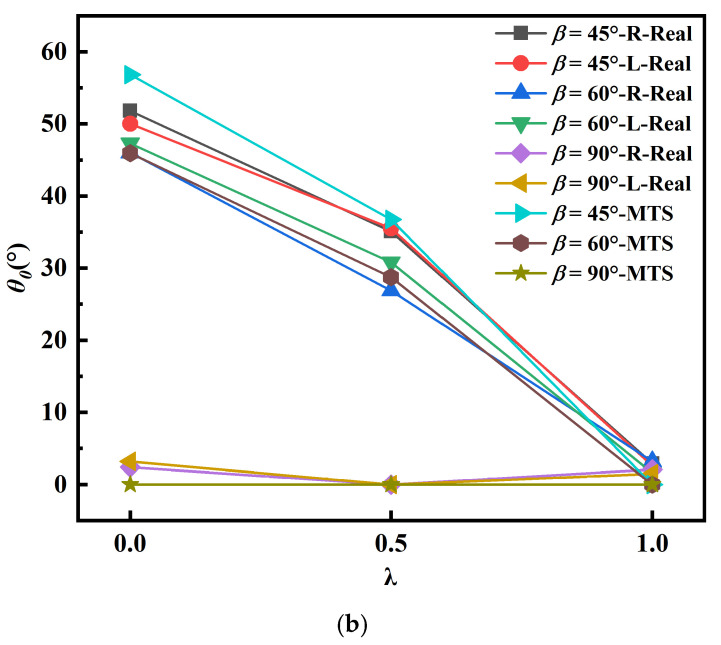
Crack initiation angle and real crack path under different biaxial load ratios. (**a**) Real crack paths from test specimens (*λ* = 0.5) and (**b**) real crack propagation angle and predicted crack propagation angle by MTS criterion.

Figure 7a is a typical picture of the crack propagation angle for biaxial load ratio *λ* = 0.5; it can be seen that the crack path during propagation is somewhat jagged and not smooth, and even that a small crack deflection phenomenon occurs. Figure 7b shows the comparison between predicted and measured propagation angles of cracks under different biaxial load ratios and inclination angles based on MTS criterion. It can be seen that MTS criterion is better for prediction of the crack propagation angle. When *β* = 90°, the crack path is extended along the initial crack direction, and when *β* = 60° or 45°, the prediction of crack propagation angle by MTS criterion is also consistent with the measured value. When biaxial load ratio is 1, the crack growth path hardly changes for mode I or mixed mode I-II crack, and it is along the direction of initial crack. This is consistent with the characteristics observed by Leevers [26].

Figure 7b shows that the crack propagation angle decreases with the biaxial load ratio. A crack initial angle becomes smaller, the deflection angle of the crack also becomes smaller. At the same time, the crack deflection direction is all toward the direction perpendicular to the maximum principal stress. In short, for a mode I crack, the propagation direction is perpendicular to the crack path, and its fatigue crack growth behavior is completely controlled by *K_I_*. Once the crack is subjected to mixed mode I-II loading, the fatigue crack growth behavior will depend on the combined action of *K_I_* and *K_II_*. It can be found that the crack propagation angle becomes larger with crack inclination angle *β* under the constant biaxial load ratio, and it can be seen that *β* has a significant effect on the crack propagation angle of mixed mode I-II fatigue crack growth. As *β* decreases, the crack propagation angle increases continuously, and the crack propagation angles are 0°, 26.9°, and 35.1° (to the left of the crack), and 0°, 30.8°, and 35.5° (to the right of the crack) for *β* of 90°, 60°, and 45°, respectively. With the variation of *β*, the propagation direction of the mixed mode I-II crack is controlled jointly by *K_I_* and *K_II_* [23]. Both Qian [27] and Richard [28] have also demonstrated the correctness of MTS criterion with experimental results.

Under uniaxial loading, the crack initiation angle with the crack inclination of 45 degree is of some error compared with the predicted value by the MTS criterion which is due to that under uniaxial loading (Y-direction) the other loading arm is constrained in the X-direction with zero load. This is somewhat different from that with uniaxial specimen. The maximum error is at 9%, and the overall average error is at 5%.

## 5. Experimental Results and Analysis

### 5.1. Fatigue Crack Growth of Mode I Crack

**Figure 8:**
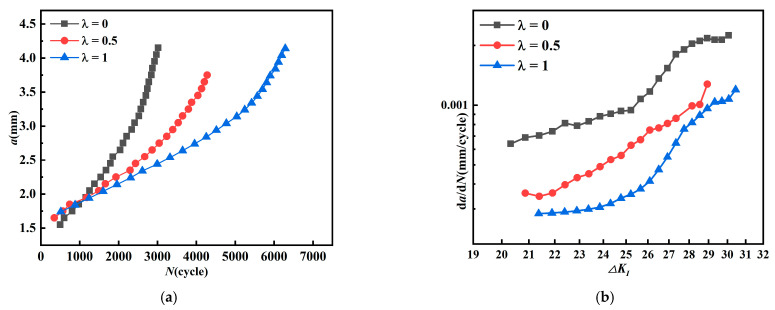
*a-N curves* and crack growth rate (*β* = 90°). (**a**) *a-N curves*. (**b**) The relationship between *da/dN* and *K_I_*.

Observing the *a-N curves* in Figure 8a, it can be found that as the biaxial load ratio increases, the crack growth life also increases. As the crack gradually propagates, the growth rate under *λ* = 0 starts to lead more than that under *λ* = 0.5 and 1, and becomes faster and faster. Combined with Figure 8b, it can be clearly seen that the crack growth rate under uniaxial loading (*λ* = 0) is undoubtedly the fastest. The load conditions under *λ* = 0.5 and *λ* = 1 start to show a clear bifurcation when the crack length reaches 2.3 mm, where under *λ* = 0.5 crack starts to show a clear acceleration, and the fatigue crack growth under *λ* = 1 is the slowest. From Figure 8b, the growth rate under *λ* = 1 is of a lower level, and the fatigue crack life is longer compared to that under *λ* = 0 and *λ* = 0.5, which is consistent with the conclusion by Lee [16] that the fatigue crack life increases with biaxial load ratio in the range 0 to 1.

When the initial crack inclination angle is 90°, *K_II_* is equal to zero, and the crack growth is completely controlled by *K_I_*; the relationship between *da/dN* and *ΔK_I_* is shown in Figure 8b. Under *λ* = 0, the crack growth rate is at a higher level, leads throughout the whole propagation process, and also become slower with the increase of *λ*. Anderson and Garrett [17] found a similar phenomenon in their study, where a change of *λ* leads to change of crack growth rate. Furthermore, compared with Figure 4, when *λ* = 0, the stress intensity factor *K_I_* is the highest and also the crack growth rate is the fastest. The curves in Figure 8b show some instability at the end of crack growth, with irregular fluctuations of crack growth rate. Breitbarth [29] observed by DIC technique that crack in the center of specimen leads to redistribution of stress and strain field during test, especially when the plastic zone at the tip of crack reaches the boundary of zone, and the crack becomes unstable. As a whole, the fatigue growth rate decreases with biaxial load ratio *λ* for mode I crack, this result is consistent with the conclusion reached by Hopper and Miller [15] that loads parallel to the crack lead to a decrease of crack growth rate.

### 5.2. Fatigue Crack Growth of Mixed Mode I-II Crack

**Figure 9:**
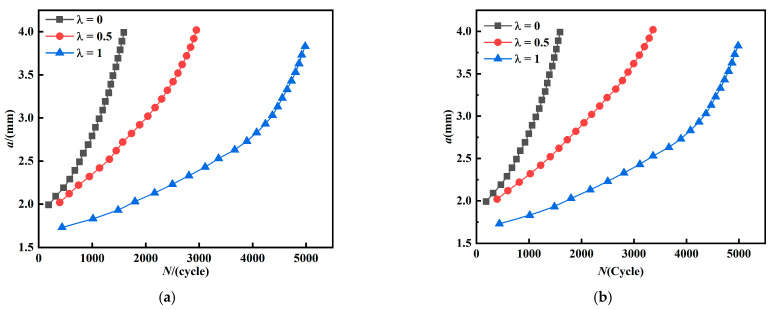
*a-N curves* under crack inclination *β* = 60°, 45°. (**a**) *a-N curves* (*β* = 60°), (**b**) *a-N curves* (*β* = 45°).

Figure 9 shows the *a-N curves* of cruciform specimens with mixed mode I-II crack (*β* = 60°, 45°), and it is also seen that the longest life and the slowest fatigue crack growth rate are found under *λ* = 1.

**Figure 10:**
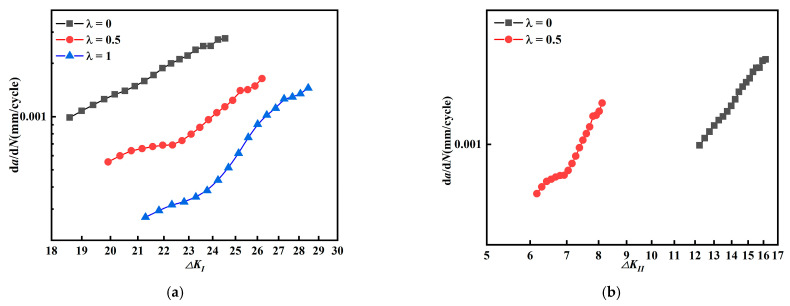
Relationship between stress intensity factor amplitude *ΔK_I_*, *ΔK_II_*, and *da/dN* (*β* = 60°). (**a**) Relationship between *ΔK_I_* and *da/dN*. (**b**) Relationship between *ΔK_II_* and *da/dN*.

Figure 10a,b show the relationship between the stress intensity factors amplitude *ΔK_I_* and *ΔK_II_*, and the fatigue crack growth rate *da/dN*, respectively, and it is found that both curves show the opposite order. In Figure 10a, the fatigue crack growth rate decreases with biaxial load ratio *λ* when the crack growth rate is related with *ΔK_I_*. In Figure 10b, the crack growth rate increases with biaxial load ratio *λ* when the crack growth rate is described by *ΔK_II_*.

**Figure 11:**
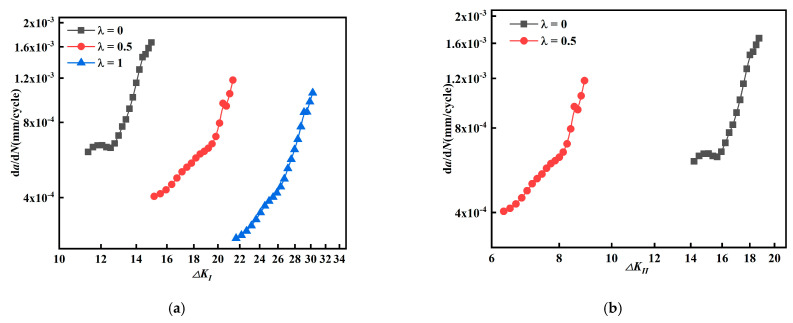
Relationship between stress intensity factor amplitude *ΔK_I_*, *ΔK_II_*, and *da/dN* (*β* = 45°). (**a**) Relationship between *ΔK_I_* and *da/dN*. (**b**) Relationship between *ΔK_II_* and *da/dN*.

Figure 11 shows *da/dN* crack growth rate at different *ΔK* (*β* = 45°). For cruciform specimens with a mixed mode I-II crack, Figure 11a plots the crack growth rate between *ΔK_I_* and *da/dN*, and Figure 11b plots the relationship between *ΔK_II_* and *da/dN*; the results are consistent with Figure 10.

Further observation of Figure 5 and Figure 6 shows that as the biaxial load ratio increases, the stress intensity factor amplitude *ΔK_I_* increases and *ΔK_II_* decreases. Comparing Figure 5 and Figure 6 with Figure 10 and Figure 11, as the biaxial load ratio increases, *ΔK_I_* increases and *da/dN* decreases; at the same time, *ΔK_II_* decreases and *da/dN* increases. It can be deduced that the fatigue crack growth rate *da/dN* does not depend on the single contribution of stress intensity factor amplitude *ΔK_I_* or *ΔK_II_*.

### 5.3. Equivalent Stress Intensity Factor and Fatigue Crack Growth

As seen above, the mixed mode I-II crack is not controlled only by stress intensity factor amplitude *ΔK_I_* or *ΔK_II_,* and it is not appropriate to use either *ΔK_I_* or *ΔK_II_* alone to describe the fatigue crack growth rate. In other words, mixed mode I-II fatigue crack growth is more complex [21].

For the cruciform specimens with a mixed mode I-II crack, both stress intensity factors amplitude *ΔK_I_* and *ΔK_II_* are present under both uniaxial and biaxial loading conditions.

Tanaka [30] used the equivalent stress intensity factor *K_eq_* to describe the relationship with fatigue crack growth rate *da/dN*.

(11) $$K_{eq}=\sqrt[{4}]{K_{I}^{4}+8K_{II}^{4}}$$

Biner [8] used the effective stress intensity factor *K_eff_* to investigate the relationship with *da/dN*.

(12) $$K_{eff}=\sqrt[{2}]{K_{I}^{2}+K_{II}^{2}}$$

As seen in Equations (11) and (12), the coefficients of *K_II_* are different. Tanaka believes that *K_II_* contributes more during crack propagation process and gives *K_II_* a higher weight. Biner believes that the weight of *K_II_* during crack growth is limited and the coefficient of *K_II_* is equal to1.

Based on equivalent stress intensity factors and an effective stress intensity factor, the contribution of *K_II_* is explored and an equivalent stress intensity factor is identified to describe the fatigue crack growth rate.

**Figure 12:**
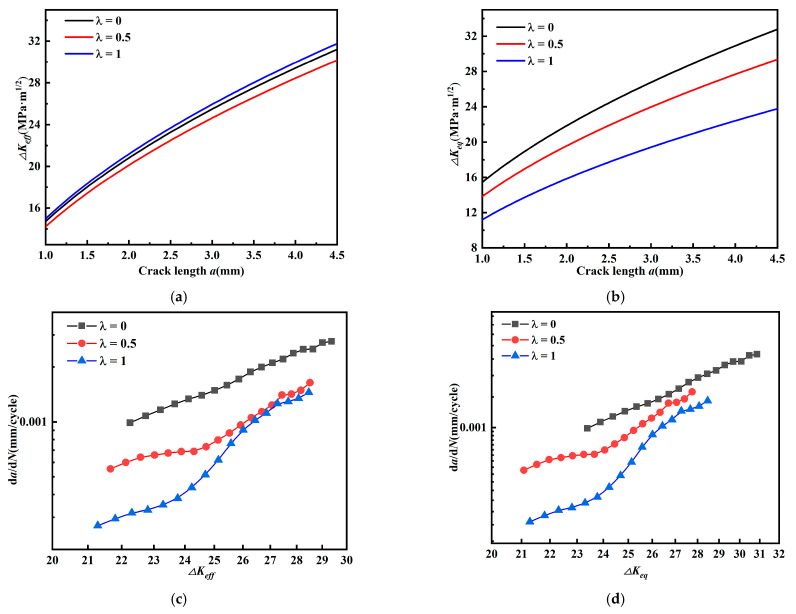
Relationship between crack length, equivalent and effective stress intensity factor amplitude, and *da/dN* (*β* = 60°). (**a**) Relationship between *a* and *ΔK_eff_*, (**b**) relationship between *a* and *ΔK_eq_*, (**c**) relationship between *ΔK_eff_* and *da/dN*, and (**d**) relationship between *ΔK_eq_* and *da/dN*.

The relationship among crack length, effective stress intensity factor amplitude, and *da/dN* (*β* = 60°) is given in Figure 12. The effective intensity factor amplitude *ΔK_eff_* (Figure 12a) does not show a regular change as biaxial load ratio *λ* increases; while equivalent stress intensity factor amplitude *ΔK_eq_* (Figure 12b) decreases as biaxial load ratio *λ* increases.

In Figure 12c,d, fatigue crack growth rate decreases as the biaxial load ratio *λ* increases. This indicates that the effect on the fatigue crack growth rate is not well described by the effective stress intensity factor amplitude *ΔK_eff_*, while the equivalent stress intensity factor amplitude *ΔK_eq_* can describe the effect on fatigue crack growth rate.

**Figure 13:**
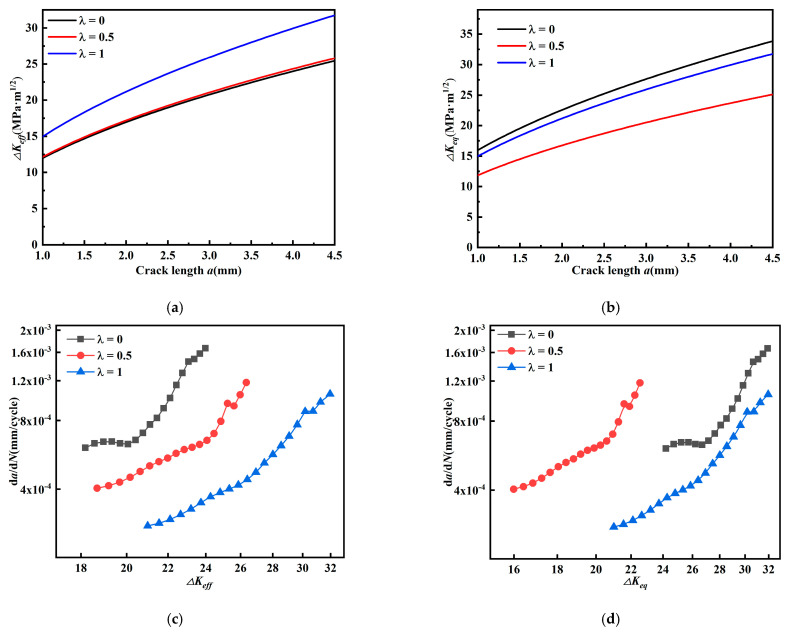
Relationship between crack length, equivalent and effective stress intensity factor amplitude, and *da/dN* (*β* = 45°). (**a**) Relationship between *a* and *ΔK_eff_*, (**b**) relationship between *a* and *ΔK_eq_*, (**c**) relationship between *ΔK_eff_* and *da/dN*, and (**d**) relationship between *ΔK_eq_* and *da/dN*.

Similarly, the relationship between crack length, equivalent intensity factor amplitude, and *da/dN* (*β* = 45°) is given in Figure 13. The effective intensity factor amplitude *ΔK_eff_* (Figure 13a) increases as biaxial ratio increases; the equivalent intensity factor amplitude *ΔK_eq_* (Figure 13b) does not show a regular variation as biaxial load ratio increases.

In Figure 13c, fatigue crack growth rate decreases with biaxial load ratio *λ*; in Figure 13d, fatigue crack growth rate changes differently with biaxial load ratio *λ*. Comparing Figure 13a–d, it is found that their regularity is contrary to each other. These results illustrate that the effect on fatigue crack growth rate cannot be described by either effective stress intensity factor amplitude *ΔK_eff_* or equivalent intensity factor amplitude *ΔK_eq_*. The results from Figure 12 and Figure 13 show that the effective stress intensity factor amplitude *ΔK_eff_* and equivalent intensity factor *ΔK_eq_* cannot effectively account for the contribution of the stress intensity factor amplitude on the fatigue crack growth rate for cruciform specimens with a mixed mode I-II crack with crack inclination angle *β* = 60° and 45° at the same time.

In addition to models of Biner and Tanaka, there are also models from Demir [31], Richard [32], and Pook [33], which are different equivalent combinations of *K_I_* and *K_II_*. They all proposed different models in their respective studies to describe the different contributions of *K_I_* and *K_II_* in crack propagation.

**Figure 14:**
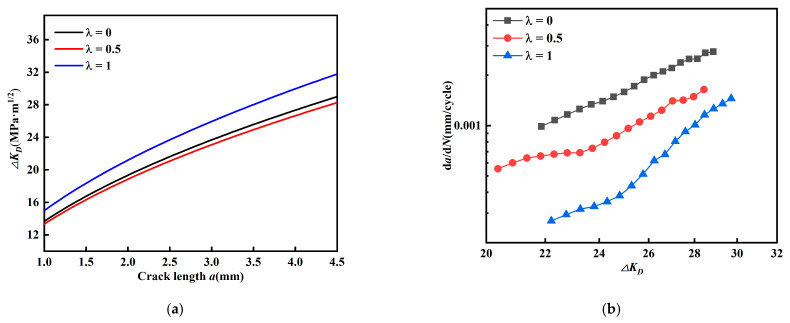
Demir model. (**a**) Relationship between *a* and *ΔK_D_* (*β* = 60°). (**b**) Relationship between *a* and *ΔK_D_* (*β* = 60°).

**Figure 15:**
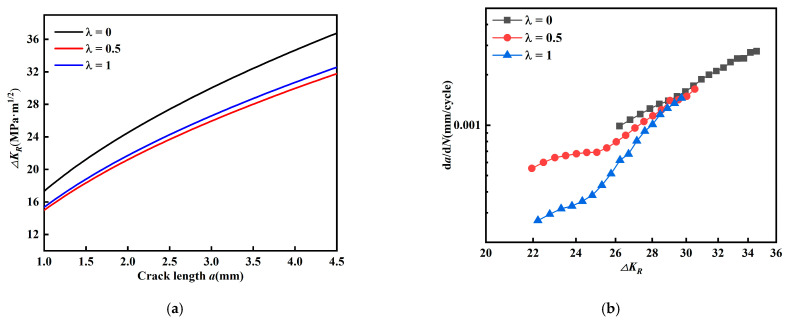
Richard model. (**a**) Relationship between *a* and *ΔK_R_* (*β* = 60°). (**b**) Relationship between *a* and *ΔK_R_* (*β* = 60°).

**Figure 16:**
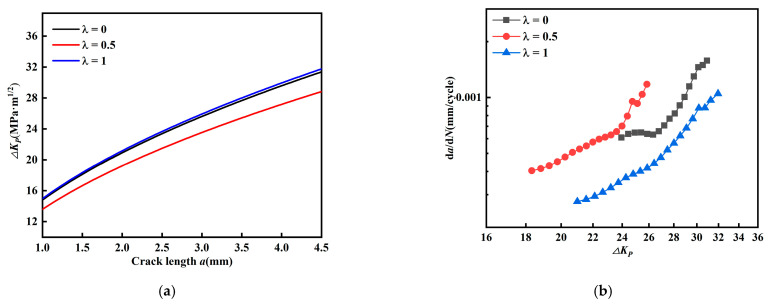
Pook model. (**a**) Relationship between *a* and *ΔK_R_* (*β* = 45°). (**b**) Relationship between *a* and *ΔK_R_* (*β* = 45°).

Figure 14 describes the fatigue crack propagation law at *β* = 60 degrees using the Demir model. The fatigue crack propagation rate should increase with the increase of propagation driving force K. However, Figure 14a,b show contradictory phenomena. The crack driving force is the largest, but the propagation rate is the smallest. Figure 15 shows the description of the Richard model for the fatigue crack propagation law at *β* = 60 degrees, and similarly there are contradictions between the two. From the Pook model in Figure 16, it can be seen that the same contradiction appears for *β* = 45 degrees. This indicates that the above model is not suitable for describing the fatigue crack propagation law in this paper. To this end, a new valid stress intensity factor *K_V_* by correcting the weight coefficients of *K_II_* is proposed here

(13) $$K_{V}=\sqrt{K_{I}^{2}+2K_{II}^{2}}$$

**Figure 17:**
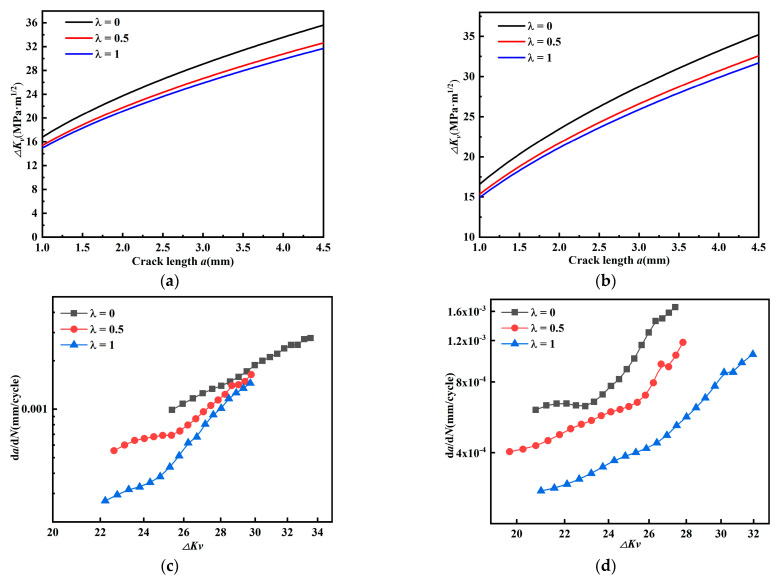
Relationship between crack length, equivalent stress intensity factor amplitude and *da/dN*. (**a**) Relationship between *a* and *ΔK_v_* (*β* = 60°), (**b**) relationship between *a* and *ΔK_v_* (*β* = 45°), (**c**) relationship between *ΔK_v_* and *da/dN* (*β* = 60°), and (**d**) relationship between *ΔK_v_* and *da/dN* (*β* = 45°).

Figure 17a,b show the relationship between the crack length a and *ΔK_v_*, and it can be found that the equivalent force intensity factor decreases with biaxial load ratio (*β* = 60°,45°). Figure 17c,d shows the relationship between *ΔK_v_* and *da/dN*, where the fatigue crack growth rate decreases with biaxial load ratio consistently (*β* = 60°,45°). Compared with *K_eff_* and *K_eq_* by Biner and Tanaka, the valid stress intensity factor *K_v_* proposed in this paper is more suitable for describing the fatigue crack growth rate of CP-Ti cruciform specimens.

In this paper, the fatigue crack propagation of commercial titanium under biaxial loading is investigated, the correction factors of the stress intensity factor suitable for small cruciform specimen is proposed, and the valid stress intensity factor model *Kv* is corrected from previous studies. The purpose is to improve the study of fatigue crack propagation of commercial titanium under biaxial loading so as to ensure the safety of pressure-bearing equipment and its structure.

## 6. Conclusions

Fatigue crack growth behavior of CP-Ti under different biaxial load ratios and crack inclination angles is investigated in this paper. Through a finite element method and biaxial fatigue crack propagate test, the following conclusions can be obtained. Based on the finite element results, the shape coefficients and biaxial load ratio factor are obtained by regression, and the modified solutions of the stress intensity factors *K_I_* and *K_II_* for the cruciform specimens are derived.

(1) The MTS criterion can well describe the crack propagation angle for mixed mode I-II crack growth and under uniaxial or biaxial fatigue crack growth. Initial crack inclination angles have a great effect on the crack propagation angle, and the crack propagation angle decreases with crack initial angle. When the biaxial load ratio increases, the crack propagation angle becomes smaller;
(2) It is found that for cruciform specimens with a mode I or mixed mode I-II crack, the fatigue crack growth rate decreases with biaxial load rate;
(3) For mixed mode I-II crack, a new equivalent stress intensity factor *K_v_* is proposed, which well explains the experimental results of fatigue crack growth rate of cruciform specimens under uniaxial or biaxial loading condition. Fatigue crack growth rate of a mixed mode I-II crack is affected by the combined effect of stress intensity factor *K_I_* and *K_II_* with different contribution.

## Figures and Tables

**Table 1:** Mechanical properties.

Young’s Modulus/GPa	Yield Strength/MPa	Tensile Strength/MPa
107.043	404	496

**Table 2:** Chemical composition (%).

Fe	C	N	H	O	Ti
0.061	0.028	0.006	0.002	0.087	99.816

## Data Availability

The data that support the findings of this study are available from the corresponding author upon reasonable request.

## Nomenclature

*a*	Crack length
*λ*	Biaxial load ratio(*σ_x_*/*σ_y_*)
*R*	Stress ratio
*β*	Initial crack inclination angle
*θ*_0_	Crack propagation angle
*N*	Number of cycles
*K_I_*	Mode I stress intensity factor
*K_II_*	Mode II stress intensity factor
*ΔK_I_*	Range of mode I stress intensity factor
*ΔK_II_*	Range of mode II stress intensity factor
*K_eq_*	The equivalent intensity factor by Tanaka
*K_eff_*	The effective stress intensity factor by Biner
*K_v_*	New the equivalent stress intensity factor
FEA	Finite element analysis
*MTS*	Maximum tangential stress
CS	Cruciform specimens
CP-Ti	Commercial pure titanium
*Y_I_(a/w)*	Mode I shape factor
*Y_II_(a/w)*	Mode II shape factor
*f(λ,β)*	Biaxial loading factor
*da/dN*	Crack growth rate
